# Rapid Patterning of 1-D Collagenous Topography as an ECM Protein Fibril Platform for Image Cytometry

**DOI:** 10.1371/journal.pone.0093590

**Published:** 2014-04-11

**Authors:** Niannan Xue, Xia Li, Cristina Bertulli, Zhaoying Li, Atipat Patharagulpong, Amine Sadok, Yan Yan Shery Huang

**Affiliations:** 1 Cavendish Laboratory, University of Cambridge, Cambridge, United Kingdom; 2 Department of Engineering, University of Cambridge, Cambridge, United Kingdom; 3 The Institute of Cancer Research, London, United Kingdom; University of California, Merced, United States of America

## Abstract

Cellular behavior is strongly influenced by the architecture and pattern of its interfacing extracellular matrix (ECM). For an artificial culture system which could eventually benefit the translation of scientific findings into therapeutic development, the system should capture the key characteristics of a physiological microenvironment. At the same time, it should also enable standardized, high throughput data acquisition. Since an ECM is composed of different fibrous proteins, studying cellular interaction with individual fibrils will be of physiological relevance. In this study, we employ near-field electrospinning to create ordered patterns of collagenous fibrils of gelatin, based on an acetic acid and ethyl acetate aqueous co-solvent system. Tunable conformations of micro-fibrils were directly deposited onto soft polymeric substrates in a single step. We observe that global topographical features of straight lines, beads-on-strings, and curls are dictated by solution conductivity; whereas the finer details such as the fiber cross-sectional profile are tuned by solution viscosity. Using these fibril constructs as cellular assays, we study EA.hy926 endothelial cells' response to ROCK inhibition, because of ROCK's key role in the regulation of cell shape. The fibril array was shown to modulate the cellular morphology towards a pre-capillary cord-like phenotype, which was otherwise not observed on a flat 2-D substrate. Further facilitated by quantitative analysis of morphological parameters, the fibril platform also provides better dissection in the cells' response to a H1152 ROCK inhibitor. In conclusion, the near-field electrospun fibril constructs provide a more physiologically-relevant platform compared to a featureless 2-D surface, and simultaneously permit statistical single-cell image cytometry using conventional microscopy systems. The patterning approach described here is also expected to form the basics for depositing other protein fibrils, seen among potential applications as culture platforms for drug screening.

## Introduction

Cell and tissue culturing has been traditionally performed on a flat, 2-D, featureless surface. In the last decade, extensive studies have revealed the profound influence of the spatial and physical properties of a substratum on cellular behavior and cell fate [1], [2]. These findings undoubtedly have opened up debates on the extent to which our present understanding, derived mainly from a 2-D petri-dish surface, is valid *in vivo*, e.g [3], [4]. 3-D culturing in a collagenous hydrogel matrix, for example, is seen as a better model system to recapitulate the *in vivo* extracellular matrix (ECM) microenvironment [5]–[7]. However, these systems require a more sophisticated imaging platform [8] not compatible with the existing high throughput systems based on 2-D image acquisition and segmentation. The variability in the gel preparation [8] may also influence the comparability of different results. Recent findings have demonstrated that cells interfacing with 1-D tracks of adhesion molecules resemble the migratory behavior in 3-D. This has opened up the possibility of designing a robust 1-D system mimicking a 3-D context [9]–[11]. This concept provides an alternative approach to investigate physiological cellular behaviors while giving a high level of compatibility with a standardized imaging platform.

Since ECMs are composed of fibrous protein structures [1], the study of cellular interaction with individual fibrils will be of physiological relevance. Each fibril not only provides the 1-D adhesive features like the cases of ref. [9], [11], but also presents the curved contour which may be experienced by cells *in vivo*. To utilize the capability of electrospinning in the efficient fabrication of bio-polymeric fibres [12]–[15], we adopt near-field electrospinning (NFES) [16], [17] to conduct fast and continuous patterning of gelatin fibrils. NFES has the advantage over traditional far-field electrospinning in curtailing the inherent electric instabilities [16], and it is employed in quest of precision control of fiber morphology, necessary to trigger well-defined cell-substrate interactions [18]. Various applications of NFES have recently been demonstrated, for instance, patterning polyethylene oxide (PEO) for integrated circuit chip insulation [16], [19], piezoelectric polymers for energy conversion [20], [21], conjugated polymers for light emitting diode [22], and carbon nanotube-PEO composites for stretchable electrodes [17]. To our knowledge, this is the first application of NFES to pattern ECM protein fibers. Its potential implementation will provide a simple, rapid and versatile alternative to the existing micro-patterning technologies such as laser writing and lithography [23].

In this study, ordered arrays of gelatin fibers were deposited onto an insulating elastomer surface over an area of tens of centimeters squared. Gelatin is chosen because it is a collagen analog [24], [25] which emulates key chemical and biological functions of a native ECM. We focus our discussion on how the solution properties of the NFES “ink” can affect the fibril morphology, at both the local profile level, and the long-range topography level. Also by discussing the underlying mechanisms affecting the solution properties, we propose general strategies to tailor-design the ECM patterns by NFES, which are believed to be of general applicability to other protein fibril systems. Finally, we test potential applications of the NFES gelatin fiber array as an imaging cytometry assay utilizing statistical analysis of cellular morphology. Since Rho-associated protein kinase (ROCK) [26] inhibition of endothelial cells has been seen as a potential therapeutic approach to reduce angiogenesis in cancer [27], we measure the morphology of EA.hy926 (an endothelial cell line [28], [29] which possess vascular formation potential in an ECM gel) in response to ROCK inhibition by H1152. The dose-dependent morphology of endothelial cells was quantitatively determined using shape parameters.

## Materials and Methods

### Material preparation and nomenclature

The NFES solution is based on an aqueous mixture of gelatin (porcine skin Sigma-Aldrich), acetic acid and ethyl acetate (Fisher Chemicals). All these materials were used without any further purification. Twelve different formulations of gelatin solutions were used in this work, as detailed in Table 1 in *Results and Discussion*. The appellation of each solution will be designated according to the following convention. The first part, with a prefix G, represents the weight percentage of gelatin; the second part, with a prefix A, represents the total weight percentage of acid and its derivative (which are mixtures of acetic acid and ethyl acetate); finally, the third part specifies the ratio of acetic acid to ethyl acetate in a bracket. For example, G8-A72(1.5) indicates a solution containing 8wt% gelatin and 72wt% acid-acyl additives, while the ratio between acetic acid to ethyl acetate is 3∶2. We also adopt the abbreviations of A.A. for acetic acid, and E.A. for ethyl acetate. To prepare the solution for NFES, gelatin was dissolved in solvents of deionized water, acetic acid and ethyl acetate at appropriate weight fractions, stirred at room temperature for 24 hours before use as a homogeneous mixture.

**Table 1:** Compositions of gelatin solutions used for NFES patterning.

Notation	Gelatin, wt%	Water, wt%	Acetic acid, wt%	Ethyl acetate, wt%	A.A.∶E.A.	Morphology
G8-A72(1.5)	8	20	43.2	28.8	1.5	none
G10-A70(1.5)	10	20	42	28	1.5	beaded
G10-A70(1)	10	20	35	35	1	none
G10-A70(4)	10	20	56	14	4	beaded
G11-A69(1.5)	11	20	41.4	27.6	1.5	beaded
G11-A64(1.5)	11	25	38.4	25.6	1.5	beaded
G11-A59(1.5)	11	30	35.4	23.6	1.5	straight
G11-A54(1.5)	11	35	32.4	21.6	1.5	straight
G11-A49(1.5)	11	40	29.4	19.6	1.5	curly
G11-A69(2)	11	40	46	23	2	beaded
G12-A68(1.5)	12	20	40.8	27.2	1.5	beaded&straight
G15-A60(1.5)	15	25	36	24	1.5	straight

The compositions of the gelatin solutions are detailed, with the global morphology of the pattern resulted for each composition also indicated.

PDMS (polydimethylsiloxane) films were used as substrates for gelatin pattern deposition where cell culturing was performed. PDMS (Sylgard 184, Dow Corning) was mixed at a 1∶10 hardener-to-resin ratio, degassed, and poured over a silicon wafer. It was then spin-coated (Electronic Micro Systems LTD, Model 4000) at 200 rpm for 120 s, and crosslinked at 60
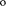
C for 3 hrs before peeling off. The PDMS substrate was measured of 660 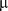
m in thickness. Prior to NFES, plasma treatment was carried out to impart hydrophilicity to the PDMS surface (Femto Science, 30 s, 25 sccm, power 10). Bovine serum albumin (BSA), purchased from Sigma Aldrich, was dissolved in distilled water to give a concentration of 0.2% w/v. 20 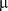
L of the solution per centimeter squared was used to coat the plasma-treated PDMS. The solution on the PDMS was then left to evaporate.

### Fabrication of a gelatin array for cellular studies

**Figure 1:**
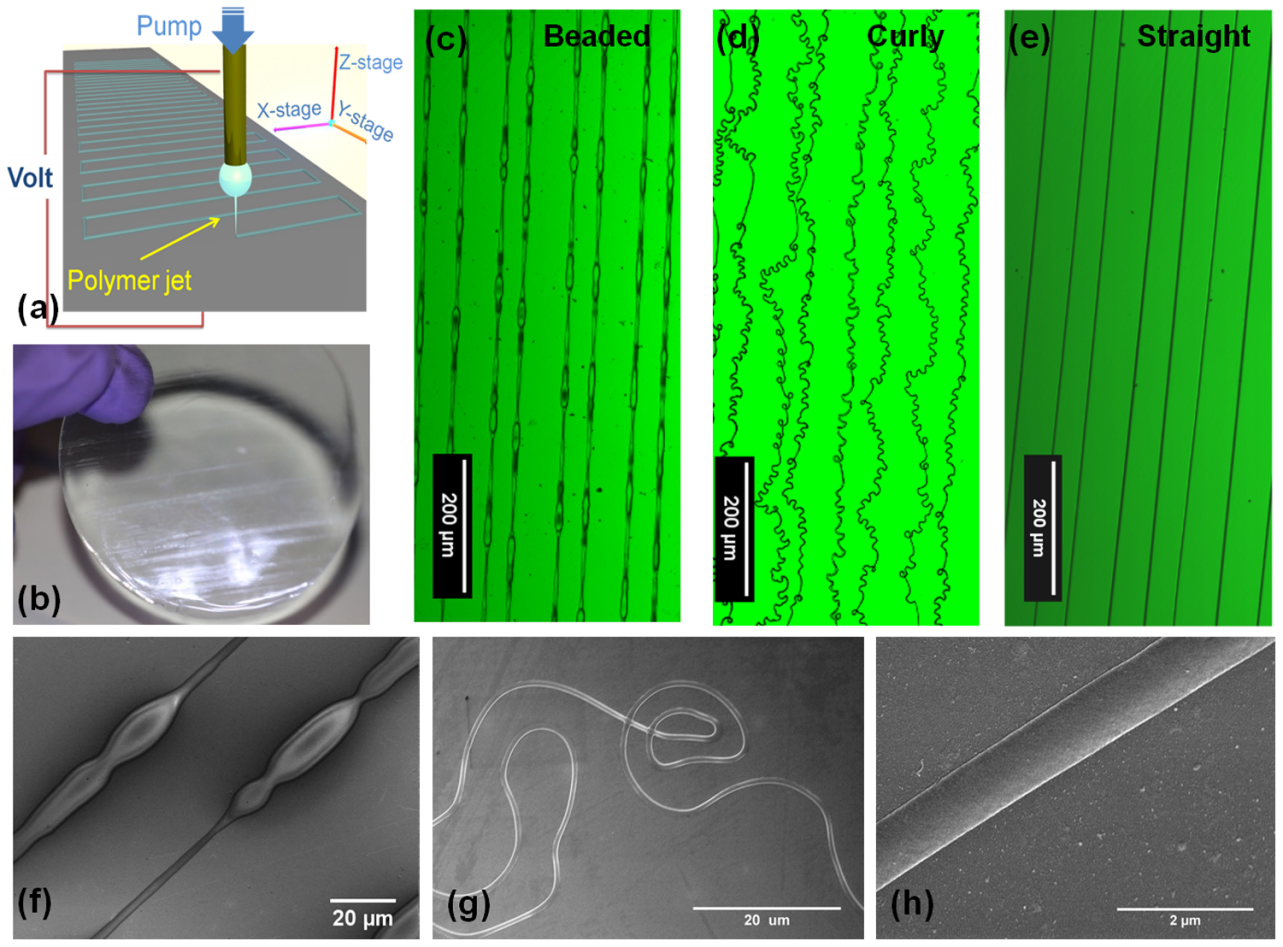
Versatile gelatin fibril patterns can be formed by near-field electrospinning over a large area. (a) Scheme of the NFES patterning process. (b) A 2 inch PDMS sheet containing arrays of gelatin patterns. (c)–(e) Confocal images of fibril patterns with bead-on-strings, coils, and uniform straight fibrils respectively. (f)–(h) Scanning electron micrographs showing typical regions of the fibril patterns illustrated in (c)–(e).

The experimental scheme of NFES is shown in Fig. 1. The set-up included a syringe pump (World Precision Instruments, AL-1000), a 1 ml syringe (BD Plastipak), a needle tip (BD Microlance, 19G), a high voltage power supply (Stanford Research Systems, INC., PS350/5000V-25W), an X-Y motion stage (PI micos, LMS-60), and a Z motion stage (Thorlabs, L490MZ/M). The syringe needle was ground blunt and its outer diameter was 1.08 mm. The anode of the high voltage power supply was attached to the syringe needle, and the cathode was connected to the grounded collector. Silicon wafers or PDMS films were used as substrates for gelatin deposition. The Z stage was adjusted so that the distance between the syringe tip and the substrate surface was 1.25 mm. Prior to patterning, a droplet of 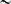
0.2 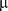
L gelatin solution was pushed out of the syringe tip using the syringe pump. Subsequently, a voltage of 1000 V was applied between the spinneret and the deposition collector to initiate an electrified jet for pattern deposition.

The cell assay used here was constructed of a gelatin fibril array of 40 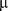
m spacing, patterned on 0.5 cm
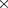
0.5 cm PDMS pieces. Crosslinking of gelatin was required to make it insoluble. After NFES, the gelatin array was immersed in a crosslinking solution formed by 25 mM of EDC (1-ethyl-3-(dimethyl-aminopropyl) carbodiimide hydrochloride, Alfa Aesar) and 10 mM of NHS (N-hydroxyl succinimide, Alfa Aesar) in ethanol/distilled water (9∶1 v/v) following the procedure by Zhang *et al.* [30]. Crosslinking was completed at 4
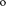
C after 24 hrs. The gelatin-PDMS construct was then rinsed with distilled water and ethanol to remove excess crosslinking agents and dried in a desiccator for 24 hr. Sterilization of the construct using 70% ethanol was applied prior to cell culturing.

### Characterization

#### NFES solution properties

In order to establish the correlation between the fiber morphology and the solution properties, we measured pH, conductivity, viscosity and surface tension of the different gelatin solutions. pH was measured using a microprocessor pH meter (pH 210, Hanna Instruments) at 26.8
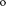
C. Viscosity was examined through a rheometer with a measuring cone CP 50-2/TG (Physica MCR 501, Anton Paar) at 20.6
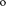
C. A fixed strain of 5% was applied, while the sweep frequency changed from the initial angular frequency of 100 rads/s to the final angular frequency of 0.1 rads/s. The conductivity of the gelatin solutions was determined using a conductivity probe (InLab 731 ISM, Mettler Toledo) at 28
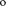
C. Finally, the surface tension of the gelatin solutions (at 24.9
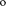
C) was deduced from the pendant drop method using ImageJ [31].

#### Physical properties of gelatin fibres

For the fiber morphology characterization, the in-plane morphology of the gelatin patterns was examined using confocal microscopy (Zeiss LSM 510), and scanning electron microscopy (SEM, XL 30 sFEG, Philips). For SEM, samples were gold coated to improve charge dissipation during imaging. To identify the local profile of a dried fibre (*i.e.* the shape of an individual stripe), atomic force microscopy (Veeco Dimension 3100, Digital Instruments) was used, with imaging performed at tapping mode to minimize surface damage.

The topology and the mechanical properties of the fibre in liquid were further characterized using in-situ atomic force microscopy (AFM) imaging and indentation based on a Nanowizard AFM (JPK Instruments, Germany). All measurements were taken in distilled water at room temperature. Preparation procedure involved fixing the sample onto a piece of mica using Araldite and inserting it into a liquid cell. The samples were pre-soaked in distilled water for over 10 min prior to measurement. In order to match the stiffness of the substrate and optimize sensitivity, a relatively soft cantilever was used. A silicon tip (NSC36, Mikromasch) was chosen with a cone half-angle of 15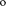. The tip spring constant was calibrated in water using indentation on mica and thermal activation. This approach gave a tip sensitivity of 28
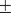
2 nm/V and a normal spring constant of 2.0
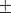
0.2 N/m. In-situ AFM imaging was performed in the intermitted contact mode (also known as tapping mode) and the force mapping was taken in the contact mode. In the force mapping measurement, the tip approached the sample with a constant speed. When the tip reached the surface, it indented the substrate until a pre-set maximum load was reached. This ensured that the indentation was controlled to be less than 100 nm which is 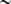
10% of the fibril thickness and should be small enough to not damage the sample material. 32
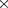
32 px^2^ indentation curves were collected on a square area. The size of the area was 10 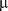
m×10 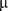
m which gave a resolution better than 300 nm
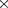
300 nm. Having adapted the Hertzian model (JPK Data Processor) [32], which was developed from the Oliver and Pharr model [33], analysis of the force curves provided the spring constant and Young's modulus of the substrate.

#### Chemical properties of gelatin fibres

Fourier transform infrared spectroscopy (FTIR) (Thermo Scientific) was used to analyze the absorption spectrum of gelatin samples, that is, uncrosslinked gelatin film, crosslinked gelatin film, uncrosslinked NFES gelatin fibrils, crosslinked NFES gelatin fibrils, and finally gelatin pellets compacted from mixtures of KBr and as-received gelatin powder. The collected absorption spectra were converted to transmission spectra using the Omnic program. The baselines were adjusted to allow comparison of the spectra after smoothing.

To evaluate whether the incubation of cells with crosslinked gelatin would alter its surface chemistry, attenuated total reflectance (ATR) infrared spectroscopy (Bruker Tensor 27 Spectrometer) was performed. Gelatin films were made by leaving drops of gelatin solutions of electrospinning grade on PDMS substrate to dry in the fume cupboard at room temperature for 24 hr. They were cross-linked according to the same procedure above for 24 hrs. The sides were flipped and cross-linked for another 24 hr to ensure homogeneous cross-linking across the film thickness. The films are then rinsed with deionised water and ethanol before storage in ethanol. For cell culturing, the films were washed in PBS, and then soaked in the cell culture medium for 30 min. Cells were directly seeded onto the gelatin film at a 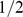 confluency and left to grow for 18 hrs (see culture conditions below). Subsequently, the cells were detached from the surface by trypsinization. After soaking the cell-detached film in deionised water overnight to remove any attached proteins, the films were then soaked in ethanol for 2 hr before drying in the vacuum oven for 1 hr. The samples were then taken for ATR scanning focusing on the surface where cells had resided. In addition to the cell-incubated samples, reference film samples subjected to the same solution incubation process without cell seeding were also studied.

### Endothelial cell assay

Human umbilical vein cell line EA.hy926 (American Type Culture Collection, Manassas, VA; passage 10–20) was maintained in Dulbecco's modified eagle medium (DMEM, Gibco) supplemented with 10% fetal bovine serum (Sigma) and 1% penicillin-streptomycin (Sigma) in 5%CO_2_ at 37°C.

When an assay was conducted, cells were seeded to obtain a coverage of 100 cells/mm^2^ on gelatin patterned PDMS, or bare PDMS (as a reference). After seeding, cells were allowed to attach and interact with the substrate for the first 12 hrs in normal culture medium. Subsequently, the media of selective samples were replaced with ROCK inhibitor-containing media (at an H1152 concentration ranging between 0.5 to 50 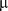
M). Incubation of another 8 hrs took place before the cell fixation. In parallel, untreated cells were maintained in normal culture medium for the entire 20 hr period before fixation.

For immunostaining, cells were fixed with 4% of paraformaldehyde solution in PBS (phosphate buffer saline) for 20 min. After being washed twice with buffer solution (0.05% Tween-20 in PBS), the cells were permeabilized with 0.1% Triton X-100 solution in PBS for 5 min. Then the sample was washed twice with the buffer again and the blocking solution (1% BSA in PBS) was applied for 30 min. Afterwards, the sample was incubated with the anti-Vinculin primary antibody (purchased from Invitrogen, used with 1∶400 dilution in blocking solution) for 1 hr at room temperature. The sample was washed three times (5 min for each) with the buffer solution. Finally, the sample was incubated for 50 min in a PBS solution containing Phalloidin (1∶400 dilution), Vinculin antibody (Invitrogen, 1∶400 dilution) and Hoechst (Invitrogen, 1/10000 dilution). The sample was then washed three times (5 min for each) with the buffer solution. Images were acquired using a Leica confocal microscope (TCS SP5 II) at randomly chosen areas of a sample, within 3 days after staining.

### Quantitative analysis of cell morphology

Morphology analysis was conducted for the fixed cell samples based on immunofluorescence images. The morphological parameters were calculated using the “Measure Object Size and Shape” module in CellProfiler software (Broad Institute, USA) [34], [35]. The cell orientation was calculated by fitting an ellipse to the cell body, so that a net cell alignment angle was obtained by measuring the angle between the cell's major axis and the fibril. We found that the software was able to correctly identify and segment both isolated and connected cells. It was able to compute the cell areas with reasonable accuracy. For example, the computed cell area for the untreated cells on bare PDMS was 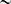
600 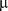
m^2^ for most of the population, which corresponded to cells of 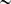
28 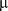
m in diameter. This compared well with the manual measurement of these approximately circular attached cells. The perimeter estimation was on the other hand less accurate and subject to underestimation since it heavily relied on the visibility of the cell edges when measured using fluorescence. Fine cellular protrusions with widths of 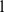 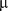
m or below were not expected to be accounted for, since our imaging condition was limited by 1 pixel (0.51 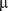
m). Finally, it is noted that we did not exclude the small number of artifacts which apparently led to results of reduction in cell areas since they did not affect the overall statistical analysis.

## Results and Discussion

### Rapid single-step patterning

For NFES of gelatin, a static droplet of gelatin solution was utilized to generate a continuous jet string for pattern deposition, see Fig. 1(a). Once electrified, the static droplet carried positive charges on its outer surface. At the bottom of the semi-sphere, where the electric field was at its greatest strength, surface cohesion was broken at a sufficient voltage level, and a polymer jet was ejected, neutralizing upon coming into contact with the collector. During continuous fabrication, a current reading of about 0.005 mA was gauged. By programming the X-Y motion stage in a regular fashion, the encroaching jet can be made to produce tailored patterns. No initiation methods such as tip probing [36] were implemented, because the edge effects of the collector produced a non-uniform and time-dependent electric field which rearranged the charge distribution and this led to shape distortion that provided the activation energy. No continuous flow [17] was used due to the precision control of the electrospinning parameters. The weight of the deposited fibril was insignificant in contrast to that of the droplet (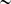
0.5% for one run), which justified the use of a static droplet. There are three benefits associated with such an ECM patterning technique. First, direct fabrication on a soft, insulating gel-like substrate is facilitated. Secondly, it presents an expeditious protocol for patterning over large areas with low material usage. It only required a single step of 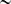
15 min to pattern a 2 inch wafer with a line density of 20 lines/mm. This incorporates much less processing steps than other existing template-based methods such as stamping or photo-patterning. Thirdly, by virtue of the versatility of electrospinning, other ECM fibrils are possible, such as elastin and laminin. An example of the gelatin-patterned PDMS is shown in Fig. 1(b).

In the absence of disconnected polymer clusters, often produced by electrospraying process [37], three distinct types of in-plane, global morphological features are displayed in this NFES work, that is bead-on-strings, coils and straight fibrils. Their optical images and scanning electron micrographs are shown in Fig. 1(c) to (e), and Fig. 1(f) to (h) respectively. It is noted that, only specific solution combinations can permit successful NFES. Solution properties tightly control both the in-plane fibril topography, and the local scale topology features. We will now discuss these effects below separately.

#### Parameter control for global patterns

**Figure 2:**
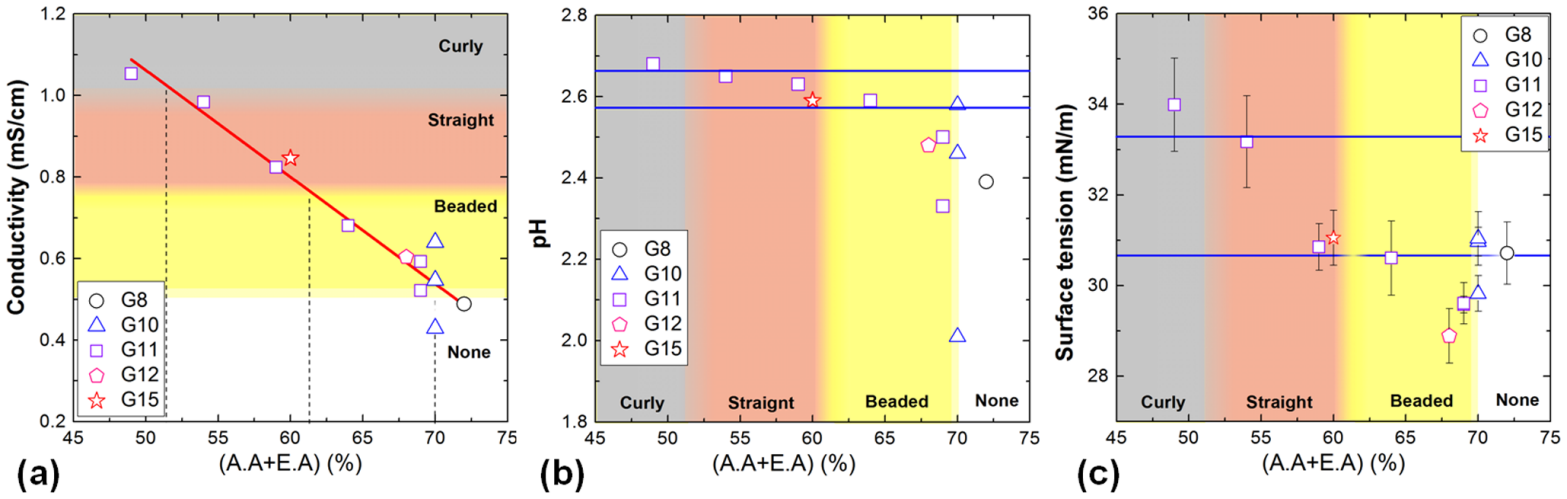
Solution properties controls global fibril morphology. Different solution properties are plotted against the total acid-acyl contents (A.A.
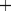
E.A.) for (a) conductivity, (b) pH, and (c) surface tension. The approximate regimes of fibril morphology are highlighted in each graph. For (b) and (c), a pair of blue lines are drawn to enclose the range of conditions which resulted in the formation of straight fibers. It is to note that for G10-A70, the A.A.∶E.A. ratios of 1, 1.5 and 4 were tested, and this can be seen as the increase in conductivity in (a).

**Figure 3:**
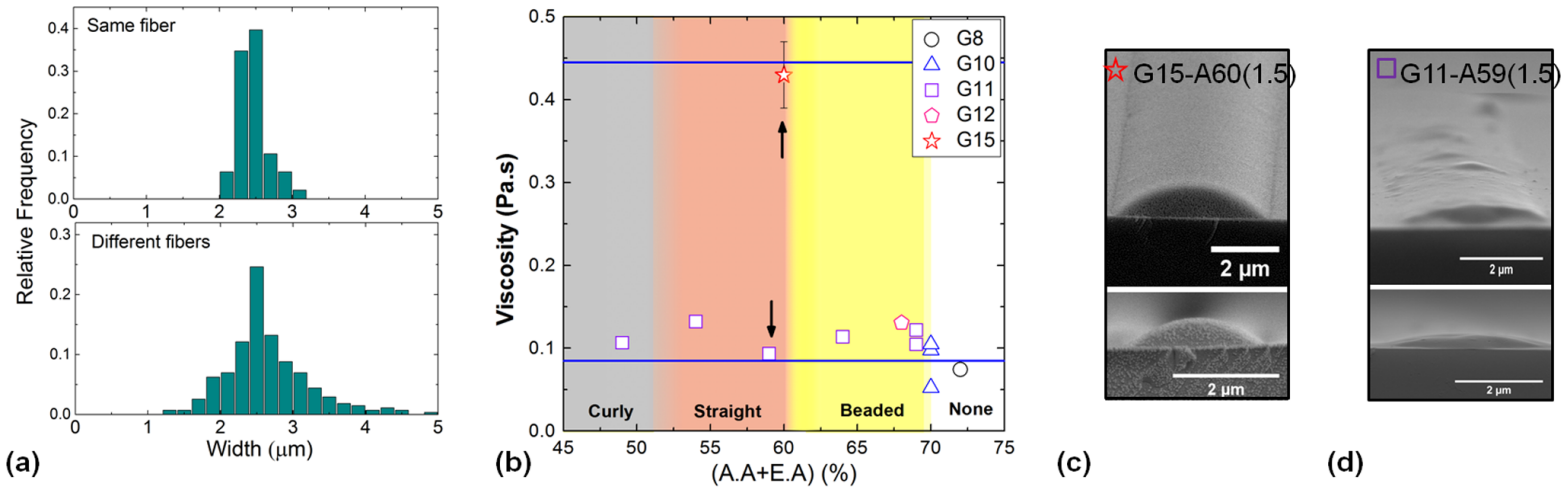
Local topology of gelatin fibril. (a) Histograms of width statistics sampled at regular intervals of a single fibril (upper panel, n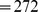), and randomly chosen positions across different fibrils (lower panel, n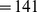). (b) Plot of viscosity against the total acid-acyl contents (A.A.
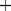
E.A.). (c)&(d) Scanning electron micrographs showing the freeze-fractured cross-section produced from solutions G15-A60(1.5), and G11-A59(1.5) respectively; their respective viscosity is indicated by an arrow in (b).

Table 1 summarizes the compositions of various gelatin solutions used in this study, as well as their resulting pattern morphology. As stated in the *Materials and Methods* section, the nomenclature of G(gelatin)wt%-A(acid-acyl)wt%-ratio is used. Analysis of the composition and patterning ability indicates that the total content of acid-acyl is the dominating factor in determining patterning ability. As the acid-acyl content increased beyond 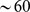
wt%, no fibrils were formed. The change in gelatin concentration also affected the solution characteristics such as conductivity, pH, surface tension and viscosity to different extents (see Fig. 2 and Fig. 3). These solution characteristics have been known to affect the performance of conventional far-field electrospinning. Here, we establish some key solution indicators for generic NFES patterning of an aqueous solution of a biopolymer.

First, by analyzing the different solution properties *vs* patterning abilities, we suggest that conductivity can be considered as the most important indicator for global morphology control (under a fixed hardware configuration). The dependence of conductivity on the acid-acyl content, *i.e.* (A.A.
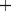
E.A.), is shown in Fig. 2(a). In general, the electrolytic conductivity decreases with the increase in (A.A.
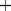
E.A.) percentage within the range of our studies. In particular, the feasibility of NFES and the three regimes of patterns can be well defined by the conductivity. NFES process would not be initiated until the solution conductivity had reached 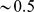 mS/cm, with the onset of beaded structures afterwards. Above 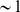 mS/cm, curly fibers formed. Hence straight fibrils were only produced within the conductivity range of 0.75 to 1 mS/cm. Now returning our focus to the solution composition, it is important to note that, both gelatin and ethyl acetate have molecular structures, whereas acetic acid has a partially ionic structure; thus acetic acid (A.A.) is the sole electrolyte out of the three solutes [38]. The electrolytic conductivity of A.A. in water is not a monotonic function of concentration. The conductivity of acetic acid *only* aqueous solution peaks at around 25 wt% following Kohlausch's Law, and subsequently decreases as the high concentration effect becomes prominent owing to more inter-ionic interaction [39], [40]. For *mixed* electrolytes, such as our systems studied here, the conductivity is approximately an arithmetic mean in terms of its constituent conductivities. Hence, given the much lower conductivity value of ethyl acetate (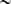
0.046 mS/cm) compared to acetic acid (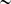
1 mS/cm) solution, the conductivity of the solution mixture approaches that of aqueous acetic acid. Thus, for a constant A.A.∶E.A. ratio, increasing (A.A.
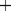
E.A.) decreases the solution conductivity. This gives rise to the general decreasing conductivity trend shown in Fig. 2(a). On the other hand, when the (A.A.
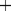
E.A.) was kept constant, the solution conductivity should increase when the E.A. weighting is decreased (*i.e.* reduction in the poorly-conductive solvent). Therefore, for the three points associated with G10-A70, an increase in the A.A.∶E.A. ratio from 1, to 1.5 to 4 had increased the conductivity across the threshold for jet initiation, and beaded fibril structures resulted. On the other hand, we observe in Fig. 2(a) that the variation in gelatin content from 8wt% (G8) to 15wt% (G15) negligibly changed the conductivity trend. In particular, both G11-A59(1.5) and G15-A60(1.5) produced straight fibrils.

Next, we focus on the dependence of fibril morphology on pH. The pH measurements for all the gelatin solutions are shown in Fig. 2(b). It seems that pH does not play an indicative role in the NFES morphology. A solution pH of 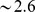 had entered all the three regimes of curly, straight and beaded fibers; though for pH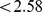, the solution resulted in either beaded fibrils or no jetting. Given the small equilibrium constant (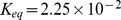) for the proton dissociation of A.A. [41], the pH value is solely determined by the A.A. mixing content. This explains the large depression in pH for G10-A70(4) (with a beaded structure at pH
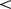
2), which had an A.A. content of 55wt%, much above the others.

We will next consider the correlation between surface tension (at the liquid/air interface) and fibril morphology, based on Fig. 2(c). The addition of A.A. (surface tension
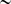
27.6 mN/m) and E.A. (surface tension
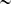
23.9 mN/m) dramatically lowered the surface tension of the gelatin solution (*cf.* that pure water has a surface tension of 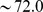 mN/m). This is in accordance with an equation proposed by Szyszkowski [42]. A trend of decreased surface tension with increased (A.A.
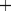
E.A.) content was first observed, but a transition in behavior took place for (A.A
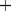
E.A) content 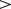
65wt%. Like pH, the surface tension does not seem to act as a good indicator for morphology control, since for example, a surface tension between 31 mN/m and 33 mN/m for a straight fiber morphology also overlapped the surface tension accompanying the no jetting regime. However, it is interesting to note that, fibers of beaded morphology were all produced by solutions of low surface tension, *i.e.* below 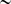
31 mN/m.

During electrospinning, the initiation of a polymer jet depends on the counteracting actions of charge repulsion and surface tension. Under a fixed electric field, the repulsive force generated depends on the number of net charges present on the surface, a factor which can be regulated by the solution conductivity. Excessive conductivity results in whipping instability (curly fibrils), whereas low surface tension leads to beading. Comparing the results of Fig. 2(a) and (c), we see that the conductivity is more strongly affected by the (A.A.
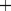
E.A.) content than the surface tension in our range of studies. In other words, tuning the solution conductivity may be regarded as a good strategy to obtain stable jetting for NFES patterning. Since the gelatin solubility depends on the A.A. content, a balance between solubility and conductivity should also be sought.

#### Parameter control for local topography

Here, we investigate the local topography resulting from NFES patterning of gelatin fibrils. For the purpose of precision patterning, our discussion will focus on straight fibrils, by measuring their widths and cross-sectional heights. As discussed previously, straight fibrils could be produced by solution G11-A54(1.5), which had a conductivity of 0.98 mS/cm and surface tension of 33 mN/m. To unbiasedly evaluate the pattern uniformity, the widths of the dry fibrils at regular intervals were measured (shown in Fig. 3(a) top panel), and the widths at randomly chosen positions across different dry fibrils were also determined (shown in Fig. 3(a) bottom panel). Both width statistics indicate an average dry fibril width of 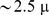
m, under the fixed hardware condition as stated in *Materials and Methods*. The distribution of widths within a single fibril is narrower than that of the cross-fibril widths. Nevertheless, more than 80% of the fibrils have dry widths within the 2–3 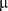
m interval. It is also expected that further optimization in the fabrication condition may reduce the variance in widths across different fibrils.

We do not observe significant variation in the average fibril width with respect to the gelatin concentration. However, the height of the fibril was significantly altered. Straight fibrils were mostly cast into a smooth parabolic silhouette, and the height stayed relatively constant along the fibril. A parabolic profile rather than a circular one might imply incomplete solvent evaporation as the jet reached the target substrate. Under this condition, solution viscosity was found to be the major factor determining the fibril height. The viscosity findings for all the gelatin solutions are plotted in Fig. 3(b). It is known that in an acidic regime, the viscosity of gelatin solution is approximately a linear function of the gelatin concentration. However, with the multiple solution components incorporated here, the gelatin concentration did not strongly affect the viscosity from 8 wt% to 12 wt%. In fact G(8) to G(12) samples had their viscosity values scattered around 0.1 Pa

s. For a 15 wt% sample G(15), a significant increase in viscosity to 0.4 Pa

s was observed. In particular, solutions G(8) to G(12) all showed a Newtonian flow characteristic, while G(15) displayed reduced viscosity with increasing rate of shear (a typical shear thinning behavior). With an increased viscosity, G(15)-A60(1.5) resulted in an aspect ratio (*i.e.* height to diameter) of 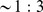, much greater than the aspect ratio of 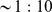 achieved by G(11)-A59(1.5), as shown in Fig. 3(c)&(d). It is noted that the high solution viscosity complicates the electrospinning process by prolonging the equilibrium settlement of the droplet into a fixed volume, during which a reduction in volume through evaporation can occur, in violation of precision control. The degree of hydration [43] of gelatin in relation to the high viscosity can be determined, with a strengthened effect in the acidic environment, in conjunction with its molecular weight and geometry [44]. Moreover, besides its subtle influence on the topology of the patterned fibrils, viscosity may also govern the mechanical properties of the resulting fibrils. This stress in viscosity over concentration has been reported recently [45].

#### Chemical and physical characteristics of NFES fibres

**Figure 4:**
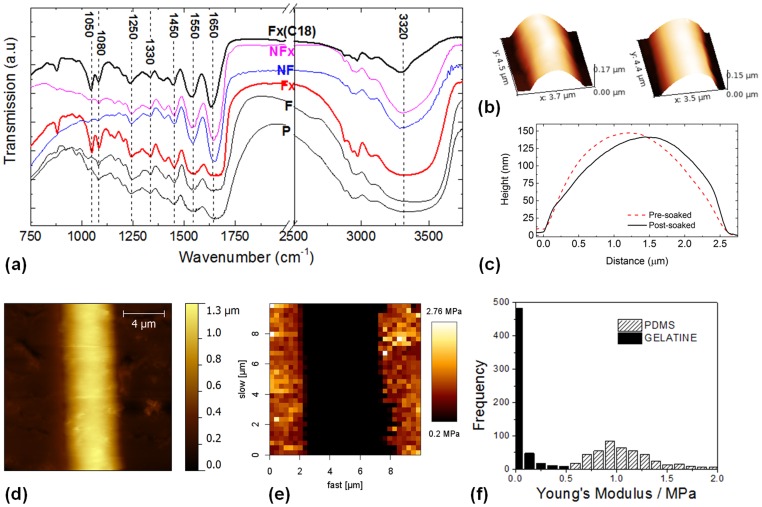
Physical and chemical properties of gelatin fibrils. (a) FTIR spectra of as-received gelatin powder (P), a cast gelatin film (F), the crosslinked gelatin film (Fx), NFES gelatin fibrils (NF), crosslinked NFES gelatin fibrils (NFx), and crosslinked gelatin films post cell culturing for 18 hrs (Fx(C18)). (b) AFM profiles of a crosslinked gelatin fibril before being immersed in water (left), and after being immersed in water for 24 hrs and dried (right). (c) Cross-sectional profile of the fibrils presented in (b). (d) In-situ AFM imaging of a hydrated gelatin fibril. (e) Young's modulus mapping of a central fibril region. (f) Histogram showing the range of Young's modulus values for the gelatin fibril and PDMS respectively.

The pH range for the NFES gelatin solutions was from 2.01 to 2.68. Gioffre *et al.* [46] have performed wide-angle X-ray diffraction analysis and demonstrated only partial denaturation of the triple-helix content of gelatin in this pH range for its integrated intensity of the 1.1-nm reflection. FTIR spectra are shown in Fig. 4(a) to evaluate the effects of NFES and crosslinking on the chemical structures of gelatin. The spectra show mostly similar peak characteristics originally present in the as-received gelatin powder [47]. The peaks that are present in all spectra include the amide A band (N-H stretching mode) detected at around 3320 cm^−1^, the amide I band (C
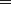
O stretching mode) at around 1650 cm^−1^, the amide II band (N-H bending mode) at around 1550 cm^−1^, the methyl bend or scissoring band at around 1450 cm^−1^, the methyl rock at around 1330 cm^−1^, the carboxyl peak (C-O stretching) overlapping the amide peak (C-N stretching) at around 1250 cm^−1^, and finally the alcohol peak (C-O stretching) at around 1080 cm^−1^ [48], [49]. For the gelatin subjected to crosslinking, we notice that the peak at 1050 cm^−1^, which belongs to ester (C-O stretching), has become more distinctive. This suggests the esterification reaction [50] between the carboxylic group and the alcohol group had taken place during the crosslinking. ATR infrared spectroscopy of the cell-incubated film surface showed unnoticeable change in the peak position or the relative peak height compared to the untreated film. Thus we suggest that the surface chemical composition of the crosslinked gelatin (as measured by a penetration depth of 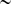
1 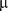
m based on typical settings of ATR), had remained similar in compositions to its original form over a period of 18 hr cell incubation. However, we do not exclude that changes to the top tens of nanometer surface had taken place. Further studies using techniques such as XPS [51] can elucidate this.

The stability of fibre geometry was also investigated by atomic force microscopy (AFM). It has been found that the crosslinking process reduced the fibril height by 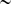
20%, but left the fibril width unchanged. Analyzing the effect of hydration on the structural integrity of the fibers, we found that the crosslinked fibril was able to retain its shape after it had been soaked for 24 hrs in deionised water and dried (see Fig. 4(b)&(c)). Furthermore, the physical property of the hydrated gelatine fibril was investigated using in-situ AFM as a reference to the physical response experienced by ECs. To interpret the force-displacement results using the Hertzian model, the following assumptions were made. (1) The indenter was rigid compared to the sample material; (2) During loading, the substrate might be deformed plastically as well as elastically. However, only the elastic component contributed to unloading. Therefore analysis was performed based on the retraction curves; (3) The material was homogeneous and isotropic and the Young's modulus was independent of the strain state; (4) The tip of the cantilever was narrow compared to the curvature of the fibril. During indentation, the fibril surface was roughly flat and loading direction was perpendicular to the surface. The Poisson's ratios of both gelatine and PDMS were assumed to be 0.5 as reported by Richards and Mark [52]. As both gelatine and PDMS are viscoelastic materials, the Poisson's ratio is highly time-dependent and can vary under different stress states [53]. In our experiment, each indentation was performed within the time scale of seconds. Thus, it can be assumed that the Poisson's ratio remained constant under such fast deformation.

In the AFM image of Fig. 4(d), the hydrated fibril is shown with a height of 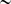
1 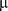
m and a width of 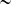
4 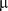
m. The increase in fibril dimension from a height to diameter ratio of 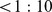 to 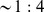, indicates the swelling of gelatine in water. In addition, the poorly defined boundary of the fibril suggests that the wet gelatine fibril has become considerably soft. This behavior is further confirmed in the Young's modulus map (Fig. 4(e)). Qualitatively, the distinct dark/bright region shows that the gelatine fibril exhibits a lower Young's modulus than the PDMS. Only the central part of the fibril has been considered for quantitative analysis. This eliminates points where the direction of indentation was not normal to the surface as well as where slipping of the tip occurred. The average Young's modulus of fibril was measured to be 226
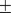
35 kPa and that of the PDMS was 1
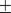
0.5 MPa. The uncertainty in the Young's modulus is up to 20%. Such deviation is contributed by both the sensitivity and spring constant of the cantilever tip. In addition, adhesion of the substrate, especially gelatine, can cause over-estimation of the contact area [54]. The standard deviation of the PDMS modulus is greater than that of the gelatine fibril. This can be caused by surface-bound BSA which can be seen in the background of Fig. 4(d). Furthermore, it is important to notice that the Young's modulus distribution (Fig. 4(f)) is approximately Poisson. This proves the results are statistically validated. Finally, the spring constant of the sample material was evaluated by analyzing the initial gradient of indentation curves. The indentation force curves show that the resultant spring constant of gelatine is 0.080
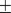
0.006 N/m and that of PDMS is 0.24
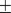
0.03 N/m. The uncertainty is approximately 10% which agrees with the uncertainty of the tip spring constant. Both spring constants are at least one order of magnitude smaller than that of the tip. This validates assumption(1) stated previously.

### Topography-guided endothelial patterns

#### Modulation of cellular morphology towards a cord-like phenotype

**Figure 5:**
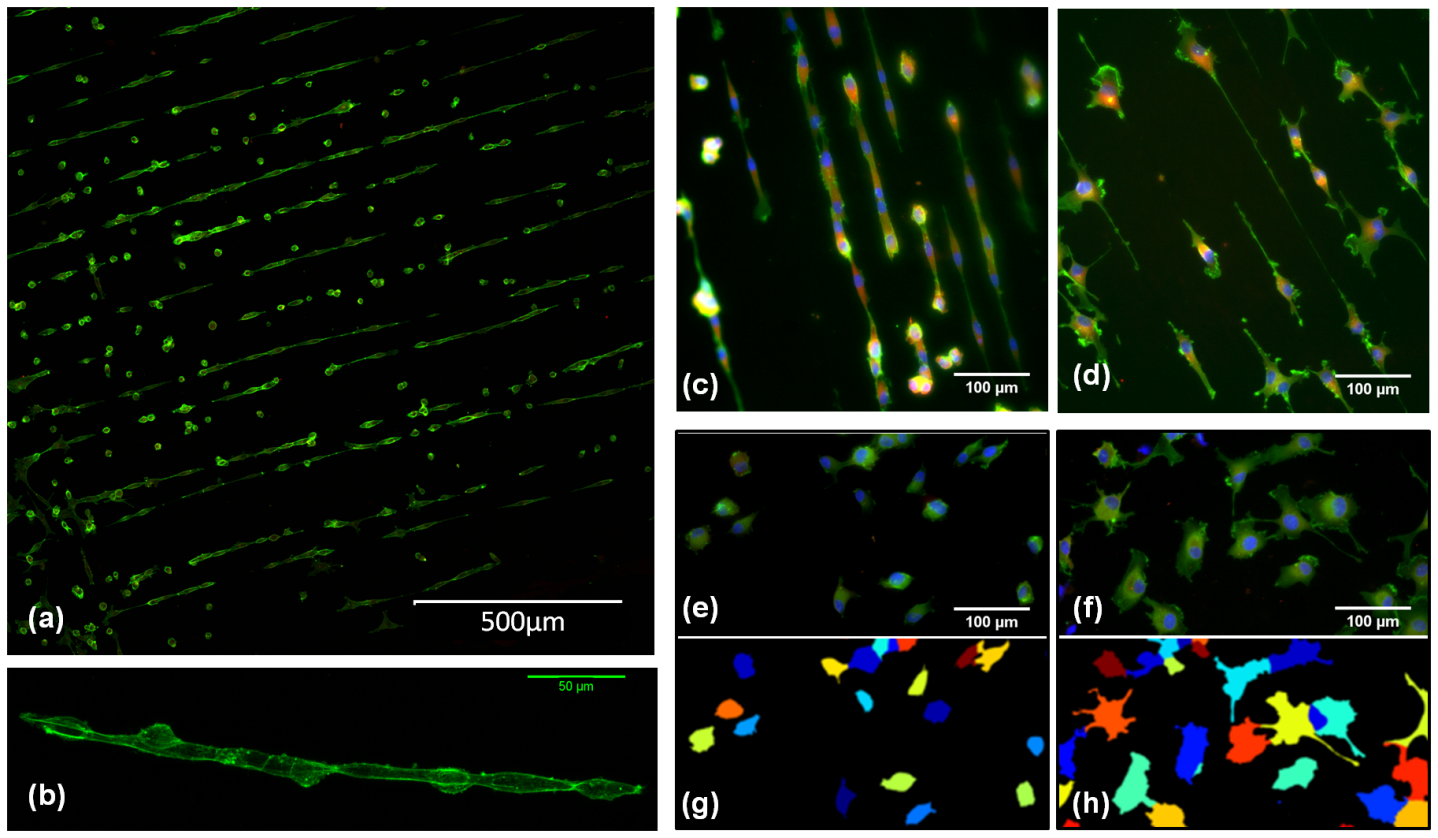
Modulation of endothelial cell (EC) pattern. (a) Assembly of endothelial “cell strings” over a large area of gelatin fibril pattern. (b) An image slice of the reconstructed 3-D conformation of a selected cell string. (c) to (f) are immunostaining images of, untreated ECs on a gelatin array (c), ROCK inhibited ECs with I(5 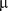
M) on a gelatin array (d), untreated ECs on bare PDMS (e), and ROCK inhibited ECs with I(5 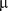
M) on bare PDMS (f); (g) and (h) are the false color segmentation images for (e) and (f) respectively. Color code for immunostaining: blue = Hoechst stain, green = Phalloidin stain, and red = Vinculin anti-body stain.

Endothelial cell line (EC) EA.hy926, known to display a number of features characteristic of vascular ECs [28], was interfaced with NFES gelatin arrays. A parallel fibril array is chosen since it is the most basic mimic of the surface topography of ECM fibrils. Benefited by the large sample area and well-defined patterns on a transparent, 2-D substrate, cell imaging was performed with a conventional inverted microscopic set-up, and multiple regions were monitored at once to obtain sufficient statistics. Figure 5(a) shows that 20 hours after plating, many of the ECs were found to form connected cell strings of a few to over ten cells on the fibril array. An image slice of the reconstructed 3-D conformation of a cell string is shown in Fig. 5(b), which illustrates the presence of actin filaments around the cell surface. Each cell string resembles the pre-capillary cords formed in a Matrigel [29] (a gelatinous protein mixture). However, we did not observe lumen formation within 20 hrs culturing according to the cell string's 3-D structure. Nevertheless, the cord-like structure is not presented on a featureless 2-D surface in the control sample, Fig. 5(e).

Various factors could trigger the cell attachment and the formation of such a cell string on the gelatin fibril. The precise mechanism does not fall in the scope of this study, though we postulate three possible reasons. Firstly, gelatin extracted from skin was used in this study, and collagen type-I was known to be the major composition of skin. Extensive studies have showed collagen type-I to initiate the angiogenesis process after the degradation of basement membrane layer (see studies summarized in a comprehensive review [55]), thus the gelatin fibril here could exhibit similar ligation sites to the EC integrins [55]. Secondly, the quasi 1-D topography of the fibril is expected to play a role in providing the physical constraints, and thus the initial cell polarization which is required for an angiogenesis process. Previous studies have also demonstrated a critical role of the adhesion topography in controlling mono-layer or cord-like morphogenesis of ECs [56]. Finally, recent studies also point to the importance of the substrate mechanical properties in controlling cell decision [57]. As shown in our mechanical measurement by in-situ AFM, the PDMS exhibits a modulus of 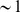 MPa, which is five times to that of the hydrated gelatin fibrils (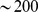 kPa). However, it is to note that previous work [56] which showed cord-like EC morphology, was based on patterning a thin layer of adhesion molecules on PDMS; thus the cells would have directly “felt” the stiffness of the PDMS interface. With the above, we can not conclude whether the ECs may prefer a more mechanically compliant substrate provided by the gelatin. Further studies in manipulating the mechanical mismatch between the fibril and the base layer may be of great interest.

To illustrate the application of such a cell assay, we investigate the morphology changes of ECs subjected to different treatments, *i.e.* normal cells or ROCK-inhibited cells cultured either on a gelatin array or a PDMS reference (see *Materials and Methods* for details). In various cell types, ROCK-dependent regulation of actin cytoskeleton enables cells' adaption to variation in the physical properties of the surrounding environment [26], [58]. Of particular interest to ECs and potential medical implications, ROCK inhibition is seen as a cancer treatment strategy to reduce angiogenesis [27]. ROCK inhibition was found to hinder the tube formation processed in tumor-derived ECs [59] and early-stage VEGF-mediated vascular formation [27] in 3-D gel; however, studies by Bryan *et al.* [27] noted that the inhibition did not affect the maintenance of an established vascular network. Since studies performed previously had shown that the morphological re-arrangement of EA.hy926 towards a capillary-like structure occurred within 12 to 16 hrs after seeding in a Matrigel [29], and a similar onset was also found on our gelatin-fibril array, ROCK inhibition by H1152 was implemented 12 hr after plating. By doing so, we suggest the experimental results are more closely related to the effect of ROCK inhibition on the capillary network forming process. To investigate the concentration effect of the inhibitor, H1152 was added at a concentration ranging from 0.5 to 50 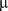
M. We use the notation of I(*concentration*), where I(5 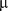
M), for example, implies an inhibitor concentration of 5 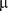
M.

Immunofluorescence images of the normal and I(5 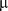
M) ECs are compared in Fig. 5(c) to (f). With I(5 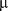
M), we observe an apparent increased area of the cell body (comparing Fig. 5(d)&(f) to (c)&(e)). The slender membrane protrusions were also much longer than the untreated cells. ROCK inhibition has been shown to promote the formation of cellular projections in a number of cell types including ECs [27]; thus our observation is in accordance with the literature report. In particular, for the fibril-interfaced, H1152 treated sample, the protrusion extension was most dramatic for the projection guided along the fibril. This dominating protrusion can reach a length over 200 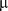
m. The long protrusions prevented close packing of neighboring ECs along the fibril, such that tightly connected cell strings are not observed. Therefore, under H1152 treatment, the ECs had lost the morphological phenotype of a capillary partially exhibited by untreated, fibril-interfaced ECs.

To quantitatively assess the observed difference described above, we utilize an open source software CellProfiler [34], [35] to segment and extrapolate the cellular morphological parameters. Combining the large, regular patterns of gelatin fibrils, and the highly adaptable image analysis procedure, a large number of cells can be analyzed from which the information of a single cell is extracted. Examples of the segmentation results are shown in Fig. 5(g)&(h). The number of morphological parameters that resulted are extensive. We choose first to investigate the correlation between the cell perimeter and cell orientation because a visible protrusion may significantly increase the cell perimeter without dramatically changing the cell area. Cell orientation is used in order to evaluate ECs' sensitivity to the geometrical/biophysical cues presented by the fibrils. Cell area is also chosen since it is one of the most commonly investigated morphological parameters, and it can also be used to validate the accuracy of segmentation (see *Materials and Methods*). We will now discuss these results in details below.

#### Image cytometry revealing interface-dependent response to ROCK inhibition

**Figure 6:**
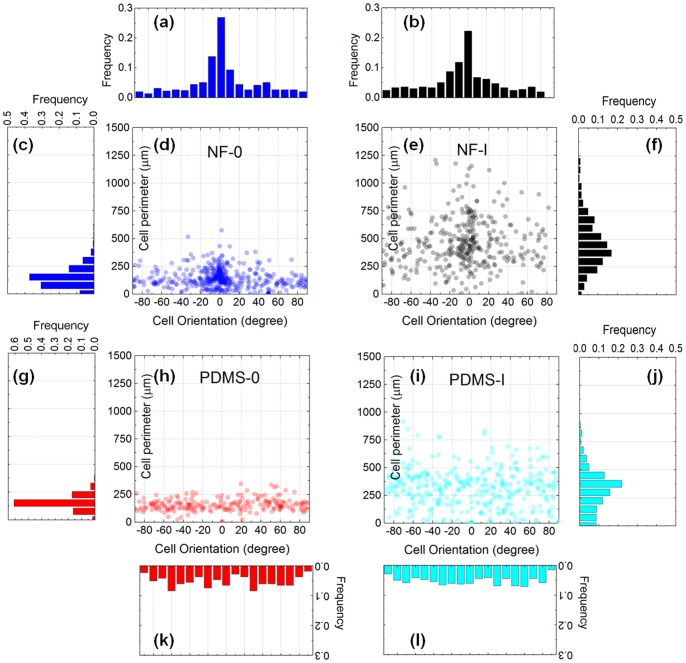
Effect of ROCK inhibition studied by single cell morphological statistics. (a),(b),(k)&(l) Histograms of frequency distribution of cell orientation for untreated cells on NFES gelatin patterns (NF-0), ROCK-inhibited cells on NFES gelatin patterns (NF-I(5 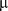
M)), untreated cells on PDMS (PDMS-0), and ROCK inhibited cells on PDMS (PDMS-I(5 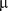
M)). (c),(f),(g)&(j) The corresponding histograms of frequency distribution of cell perimeters. (d,e,h,i) The corresponding scatter plots of the cell perimeter *vs* cell orientation.

Figure 6 illustrates the scatter plots of the cell perimeter *vs* cell orientation, for the four experimental conditions studied here. First, by comparing the histograms of the perimeter, Fig. 6(c),(f),(g)&(j), it is apparent that I(5 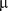
M) has dramatically increased the cell perimeter. The modal perimeter (the most likely occurring perimeter) has increased from 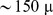
m for the untreated cells, to 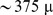
m for the inhibited cells. It is important to note that the presence of fibrils did not affect the most probable perimeter associated with cells subjected to the same treatment, *i.e.* from comparing (c)&(g) or (f)&(j); but a higher proportion of cells exhibited increased perimeters. In other words, discounting the effect of ROCK inhibition, cell polarization may be the main factor contributing to additional cell perimeters. Secondly, comparing the histograms of the orientation, Fig. 6(a)&(b) *vs* (k),&(l), we find that the gelatin array had induced EC alignment with respect to the fibril axis. For untreated ECs, 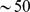
% of the cells had their major axes within 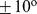 from the fibril axis, and this was comparable to the I(5 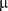
M) counterpart with 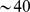
% of the population. Finally, scrutinizing the scatter plots of Fig. 6(e)&(i), we observe that the extremely large perimeter values are centered around the 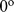 orientation in Fig. 6(e).

**Figure 7:**
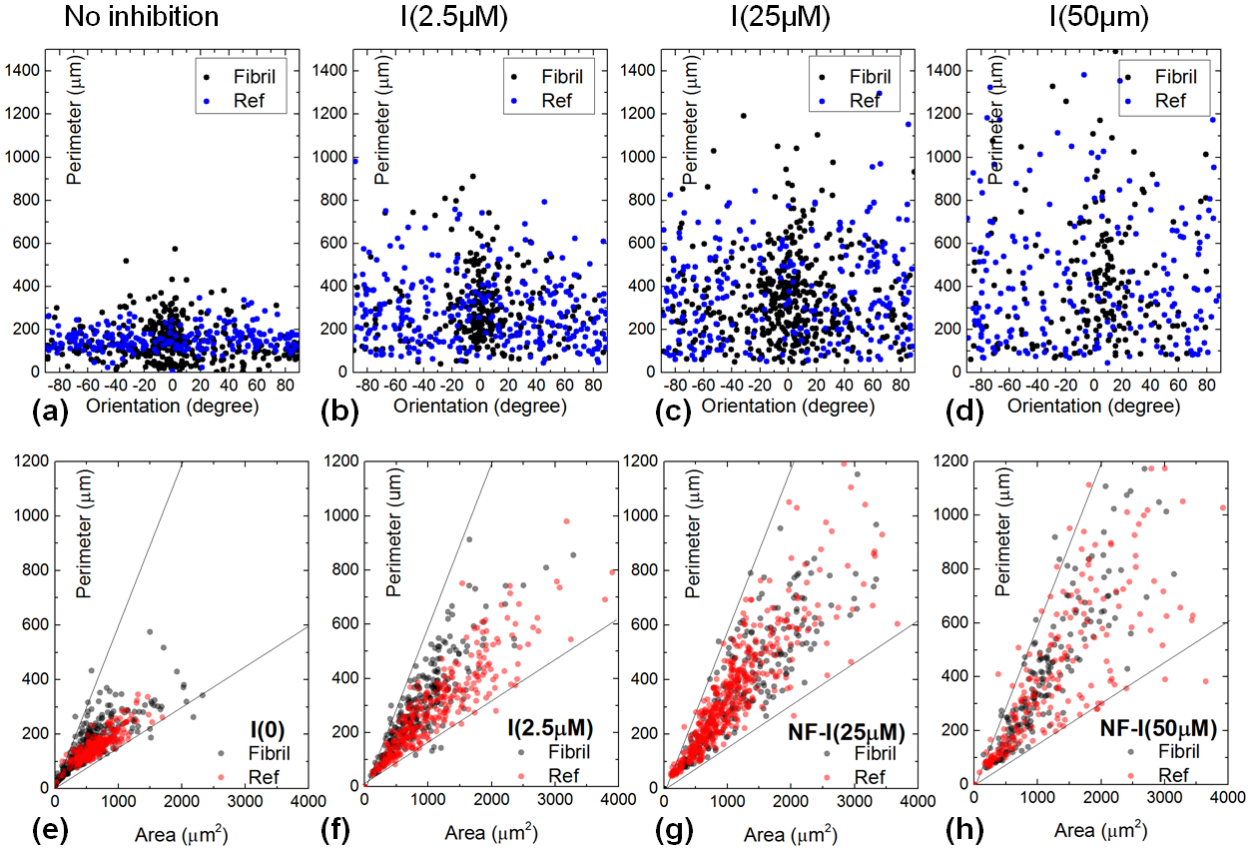
Dependence of cell morphology w.r.t. H1152 concentration. (a–d) Scatter plots of cell perimeter *vs.* orientation for ECs subject to no ROCK inhibition, and H1152 concentration at 2.5 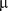
M, 25 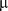
M, and 50 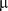
M respectively. (e–h) Scatter plots of cell perimeter *vs.* area for ECs subjected to the same treatment.

To evaluate the H1152 concentration dependence of the EC morphology, similar scatter plots of the cell perimeter *vs.* cell orientation were also produced for other treatments shown in Fig. 7(a–d). One clearly observes an increasingly wider distribution of the scatter plot w.r.t H1152 concentration. This large deviation, as a signature of the cell's reduced ability to control its shape, confirms the literature reports [58], [60]. Moreover, the cell perimeter *vs.* the cell area was also plotted which gives information on the cell's strategy to adapt its shape to a substratum. As shown in Fig. 7(e–h), their dependence was mostly confined between two boundaries, *i.e.* slopes of 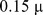
m^−1^ (lower bound) and 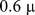
m^−1^ (upper bound). It also seems that the cell perimeter *vs.* cell area dependence moves from the lower bound to the upper bound with increasing ROCK inhibition. However, ECs' ability to sense the insoluble biochemical factors (*i.e.* presented by the gelatin fibrils) was still retained. This is based on the fact that the cell orientation is still centered at 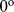 in Fig. 7(a–d) for a H1152 concentration up to 50 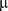
M. If the cells were to lose its ability to follow the fibrils, the scatter plot would have reversed to the form similar to that of the reference. It is also important to note that such a cellular sensitivity towards biophysical cues can only be probed by a fibril-interface, not a conventional 2-D assay. In comparison with a 3-D matrix, the unique benefit of the NFES fibrillar array lies in the ease of microscopy and image analysis, such that quantitative study of morphology phenotypes can be performed at a single-cell level.

**Figure 8:**
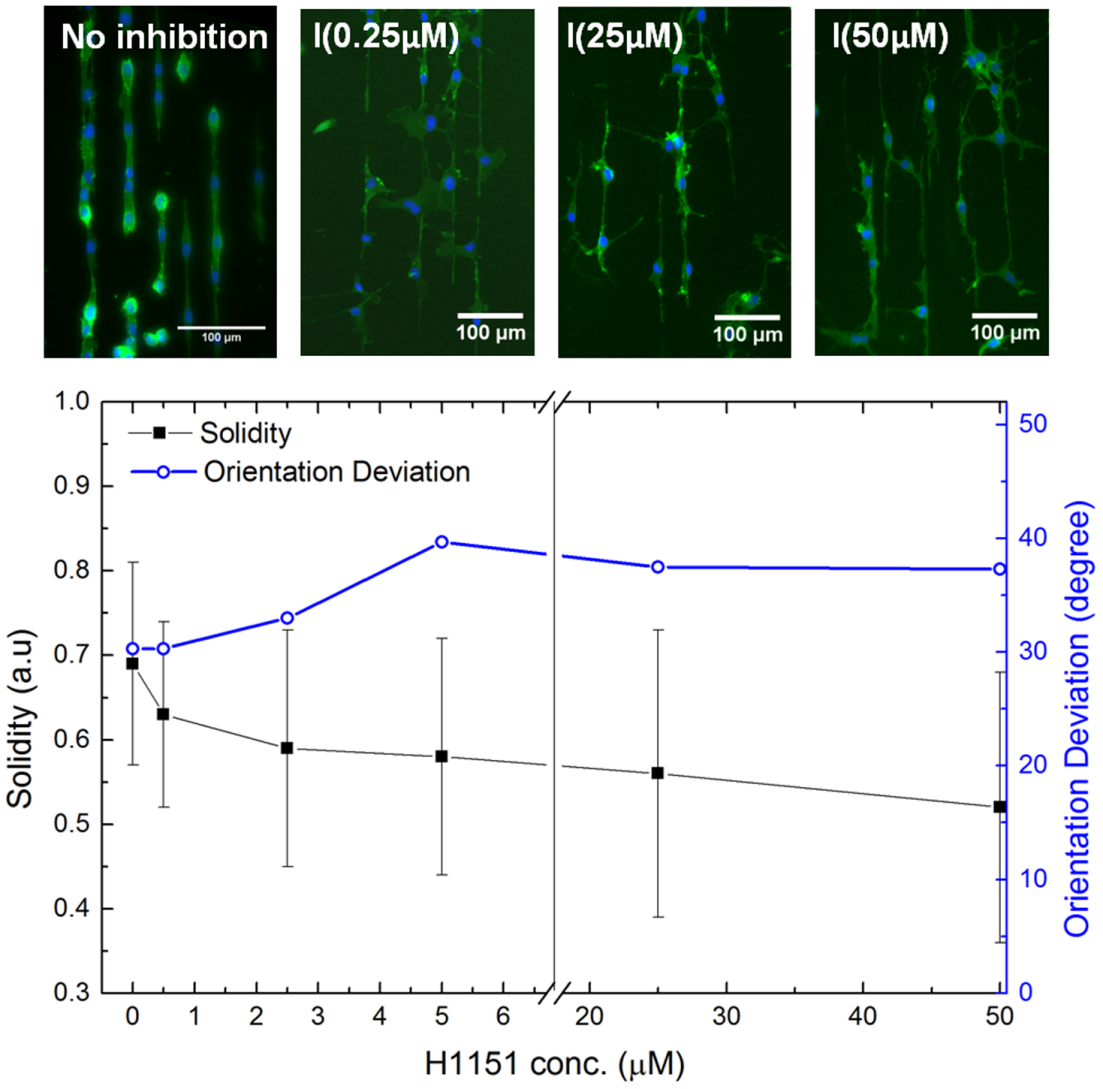
Fibril assay elucidating the drug effectiveness of H1152. Plot of 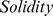 and *Orientation Deviation* w.r.t. H1152 concentration for the ECs interfaced with the gelatin fibril assay. Immunofluorescence images for the selected inhibition experiments are included as examples.

To further illustrate the potential use of such a fibril assay, we adopt two indicators to reflect the H1152 drug effectiveness. These are the shape indicator of “*Solidity*” to reflect the protrusion phenotype, and the sensitivity indicator of “*Orientation Deviation*” to reflect the cells' ability to follow the fibril's adhesive features. Here 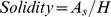, where, 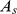 is the area of the shape region and 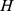 is the convex hull area of the shape. 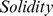 is a parameter automatically generated in the results of the “Measure Object Size and Shape” module in CellProfiler, and for a solid object of convex/round shape, 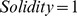. The rationale behind choosing this shape parameter as an indicator is because an added protrusion can be seen as incorporating a pronounced concave outline to the cell shape, making 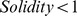. The *Orientation Deviation* is the standard deviation of the orientation angle measured from the fibril axis. For a population of randomly oriented objects taking 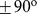 on 2-D, the mean orientation will be 0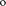 while the *Orientation Deviation* 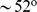 by solving 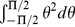. The dependence of 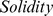 and *Orientation Deviation* on the H1152 concentration is shown in Fig. 8. The 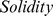 indicator decreases w.r.t the inhibitor concentration, which is in accordance with visual interpretation of the immunofluorescence images. The most rapid 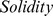 decrease occurs for H1152 concentration 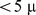
M, whereas further increase in inhibitor concentration only sees slow decrease in the 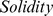 values. It is noted that the large standard deviation of the 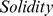 values, which is especially prominent for a high inhibitor concentration, is inherent in the shape variability rather than being induced by uncertainty/errors. For the *Orientation Deviation*, one observes that the greatest increase in values again occurs for H1152 concentration 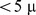
M. Beyond 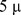
M, increasing H1152 concentration insignificantly alters the *Orientation Deviation*. The upper value of *Orientation Deviation* is 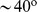 compared to the random limit of 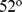. Thus even under strong H1152 inhibition treatment, the interaction between the ECs and the substrate remains. Since the most apparent changes in the shape indicators occur in the H1152 concentration 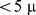
M, future studies may focus on this region for more detailed investigations.

## Conclusion

This study reports a reliable method of contriving uniform patterns of gelatin fibrils of different morphologies *via* near-field electrospinning. Conductivity is identified, among the assorted solution properties, as the most influential factor with its optimum threshold of 0.75 mS/m to yield a regular, straight pattern of gelatin mesh with tuneable line spacing through stage control. FTIR peaks, which belong to ester C-O stretching, suggest that the insoluble effect of crosslinking can be attributed to esterification of the gelatin molecules. The Young's modulus of the hydrated fibril was measured to be 226
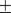
35 kPa, approximately five times softer than that of the PDMS base. PDMS sheets with the NFES gelatin fibrils mimicking aligned arrays of extracellular matrix fibrils were tested with endothelium cells EA.hy926, which are found to be promoted to form connected cell strings of capillary-like structures on the fibrils. To quantitatively reflect their morphology, the correlations between the cell area, cell perimeter, orientation and solidity are studied. Based on this gelatin fibril assay, it is found that ROCK inhibition reduced ECs' contractility underlying shape regulation, but did not diminish their ability to sense the insoluble biophysical cues directed by the extending protrusions. The fibril array is shown to present a more physiologically relevant interface to elucidate cellular behaviors than a featureless 2-D surface. At the same time, it facilitates much simpler standardized image analysis processes than a 3-D matrix, thus providing a potential materials platform for high throughput image cytometry for applications in drug screening and pathology testing. In addition to process simplicity and versatility, near-field electrospinning (NFES) patterned fibrils also produce a unique curved fiber cross-section rather than a flat adhesion molecule layer like stamping and photo patterning. Further investigations based on varying solution viscosity over a wider range than in this study can be conducted to relate cell behavior to the detailed fibril cross-section topology variations. Tuning the mechanical properties of fibrils by varying the crosslinking density can also provide a tool to investigate cell response to the stiffness of single fibrils. The method described here is believed to facilitate the patterning of a wide range of ECM relevant protein fibrils, as well as other pathological fibril assemblies such as amyloid fibrils.
